# Tear Ferning Test and Pathological Effects on Ocular Surface before and after Topical Cyclosporine in Vernal Keratoconjunctivitis Patients

**DOI:** 10.1155/2018/1061276

**Published:** 2018-10-14

**Authors:** Marcella Nebbioso, Marta Sacchetti, Guia Bianchi, Anna Maria Zicari, Marzia Duse, Paola Del Regno, Alessandro Lambiase

**Affiliations:** ^1^Department of Sense Organs, Sapienza University of Rome, p. le A. Moro 5, 00185 Rome, Italy; ^2^Department of Pediatrics, Faculty of Medicine and Odontology, Sapienza University of Rome, p. le A. Moro 5, 00185 Rome, Italy

## Abstract

**Background:**

Vernal keratoconjunctivitis (VKC) is a rare ocular surface inflammatory disease that affects mainly boys in the first decade of life. Clinical observations show that it generally regresses spontaneously with the onset of puberty, but therapeutic measures must be taken before then to control the course of the disease.

**Purpose:**

To evaluate the role of the lacrimal mucous component in VKC patients and compare tear ferning test (TFT) modifications, MUC5AC levels in tears, and density of conjunctival goblet cells to clinical characteristics before and after treatment with cyclosporine A (CY) in eye drops.

**Methods:**

Forty-seven patients affected by VKC and 30 healthy subjects aged between 3 and 16 years of life were enrolled. All individuals were submitted to complete eye examination and skin prick test (SPT) for the most common allergens. Then, they were subjected to collection of the tears and to impression cytology to evaluate TFT, MUC5AC levels, and conjunctival goblet cell density, before and after treatment with CY in eye drops.

**Results:**

Comparing the VKC group vs. the control group at baseline, a significant alteration in the degree of the ferns was found, indicating a pathological condition of the lacrimal mucous layer. In addition, an increased number of goblet cells were observed in the patients. The concentration of lacrimal secretory mucins (MUC5AC) did not show significant differences between the 2 groups. Patients treated with CY have reported improvements of some signs and symptoms of disease activity, including TFT, and a tendency of conjunctival goblet cell density to normalise.

**Conclusions:**

The results obtained demonstrated for the first time a significant alteration of the lacrimal mucin component evaluated in the VKC group, and an improvement of the latter after CY therapy.

## 1. Introduction

Vernal keratoconjunctivitis (VKC) is an ocular chronic inflammation that mainly affects children (males/females = 3/1) in the first decade of life and tends to regress generally in puberty [1, 2]. This form is relatively rare and is more frequent in warm, dry, and windy climates, such as the Mediterranean, Central Africa, India, and South America. Type I hypersensitivity linked to immunoglobulin E (IgE) and mediated Th2 lymphocytes appears to be, in fact, only one of the cofactors of the disease in which environmental, hormonal, hereditary, and immune-allergological factors interact; so, the etiopathogenesis of this disease is multifactorial [2, 3]. The typical ocular symptoms are conjunctival hyperemia, itching, tearing, photophobia, and foreign body sensation. The recent literature distinguishes this disease into 4 types [3–5]:

1. Tarsal form: small, medium, and/or giant papillae on the tarsal conjunctiva are visible with the eversion of the upper eyelid
2. Limbal form: nodules or Horner–Trantas dots are present on the corneal limbus
3. Corneal form: alterations of the corneal stroma and nerves are associated with intense hypersensitivity and photophobia
4. Mixed form which has intermediate characteristics of the 3 types

Though called “vernal”, VKC often has a chronic course throughout the year with seasonal outbreaks, in spring and summer, characterised by a worsening of symptoms, and frequent corneal involvement, ranging from superficial punctate keratitis with corneal ulcers that compromise visual function [3–5]. Diagnosis is based on clinical features, age, familiarity, disease progression, and the presence of typical clinical signs and symptoms. Allergic mediators such as histamine, leukotrienes, and prostaglandins directly stimulate secretory activity of conjunctival goblet cells, while inflammatory cytokines may regulate the secretion, proliferation, and apoptosis of the many cells involved. In fact, alterations in the expression of mucins on the ocular surface are associated with numerous pathologies, and it has been observed that a reduction or a dysfunction of the conjunctival caliciform cells results in instability of the tear film [6, 7].

In particular, several transmembrane mucins (MUC1, MUC4, and MUC16), and secretion mucins have been identified (MUC2 and MUC5AC) on the ocular surface [6–8]. MUC5AC is the major secretory mucin of the tear film, and its concentration in human tears ranges from 1 to more than 200 ng/mL. Moreover, the production of MUC5AC by the goblet cells is a target of regulation for allergic and inflammatory mediators produced by innate and adaptive immunity cells. Secretory mucous membranes remove allergens and pathogens from the ocular surface and play an important lubricating activity in epithelial cells. In fact, in patients with VKC, there is an increase in the number of conjunctival goblet cells; following the use of antihistamines and anti-inflammatory topical drugs with VKC, there is a reduction in the number of conjunctival caliciform cells to normal values [9–11]. Moreover, the expression of MUC5AC mRNA derived from goblet cells is reduced in eyes with corneal shield ulcers. At the same time, MUC1, MUC2, and MUC4 expression increases as a possible defense mechanism to compensate for the reduced protective effect of MUC5AC [8–10].

Cyclosporine A (CY) is a neutral, hydrophobic, cyclic polypeptide calcineurin inhibitor which was first purified in 1979 from cultures of the fungus *Tolypocladium inflatum* gams and from other imperfect fungi. Topical CY does not penetrate ocular tissues due to its poor water solubility and acts on conjunctiva as an immunomodulator, causing a reduction of inflammatory cells, particularly T cells, HLA-DR+ cells, and plasma cells [5–7, 12].

The purpose of this study was to evaluate the role of the lacrimal mucous component in VKC patients. In particular, the mucous component of the tear film was evaluated by tear ferning test (TFT), dosing of MUC5AC levels, and density of conjunctival goblet cells. In addition, modifications of TFT, MUC5AC levels in tears and conjunctival goblet cell density were compared to clinical features and evaluated prior and after treatment with topical CY.

## 2. Materials and Methods

We enrolled 47 children (38 males and 9 females) between 3 and 16 years of age with clinical diagnosis of VKC. They were submitted to ophthalmology clinic study by the Department of Allergology and Pediatric Immunology of the “Sapienza University of Rome”. Also, we enrolled 30 healthy controls (20 males and 10 females) aged 5–16 years. Inclusion criteria for the children in the study were as follows:

- Diagnosis of VKC based on the child's history and the presence of some signs and symptoms, including itching, photophobia, and tearing in the spring/summer
- Age between 3 and 16 years
- Symptoms associated with the presence of tarsal conjunctival papillae and/or limbal nodules, and mucous secretion
- Persistent or recurrent symptoms of VKC with little or no response to common pharmacological products for allergies
- No use of contact lenses

Exclusion criteria were as follows:

- Diagnosis of ocular pathologies such as glaucoma, blepharitis, ocular infections, cheratoconus, iritis, cataract, and other corneal or conjunctivitis pathologies
- Diagnosis of systemic pathologies such as cancer, arteriosa hypertension, diabetes, cardiophathies, etc.
- Ocular surgery in the six months prior to inclusion in the study
- Systemic or local therapy for other pathologies

In accordance with the Helsinki Declaration, all parents were informed about the use of their data, and informed consent was obtained. The study also fully complied with the Good Clinical Practice guidelines and was approved by the Ethics Committee of our Hospital, Sapienza University of Rome (Authorization Rif. CE: 2336/26.01.2012-23/11/2015).

For each patient, the following anamnestic data were collected: age, sex, family history of atopy, and autoimmune diseases, presence of associated atopic conditions, age of VKC onset, perennial or seasonal course, and prior topical therapy. All patients underwent a skin prick test (SPT) for the most common inhaled and food allergens: dog and cat epithelium, parietary, grass and olive pollen, dust mites, pine, birch, plane and cypress, cow's milk proteins, eggs, fish, wheat, and soy. Patients were also subjected to a complete eye examination with visual acuity measurement, front and back biomicroscopy, indirect ophthalmoscopy, and a clinical technique to evaluate the ocular tear film by the Schirmer I test, break-time test (BUT), fluorescein test, etc. Some clinical signs and symptoms of pathological activity to baseline were evaluated with a score from 0 to 3. The total score of the signs (TSS) was calculated as the sum of the score of conjunctival hyperemia and chemosis, conjunctival serous and mucosal secretion, large and small tarsal papillae, limbal dots or nodules, and superficial wound corneal epithelium or corneal ulceration, obtaining a TSS from 0 to 18. The total score of the symptoms (TSyS) was calculated as the sum of the score of itching, photophobia, tearing, and foreign body sensation obtaining a TSyS from 0 to 12. The tears and conjunctival cells of the patients were collected to perform: TFT, impression cytology, and MUC5AC levels. All investigations were carried out by the same physician. In the past, 33 patients (70.21%) had previously been treated with antiallergic and/or anti-inflammatory topical therapy, but without symptom control except for short periods. All patients were prescribed a multidose bottle of galenic preparation containing 1% CY in 15 mL of 0.1% sodium hyaluronate to be applied 3 times/day (Vismed Light®, Medivis Srl Catania, Italy, and 1% CY). Patients were re-evaluated 3 months after the start of topical therapy.

### 2.1. Procedure

#### 2.1.1. Tear Ferning Test (TFT)

**Figure 1:**
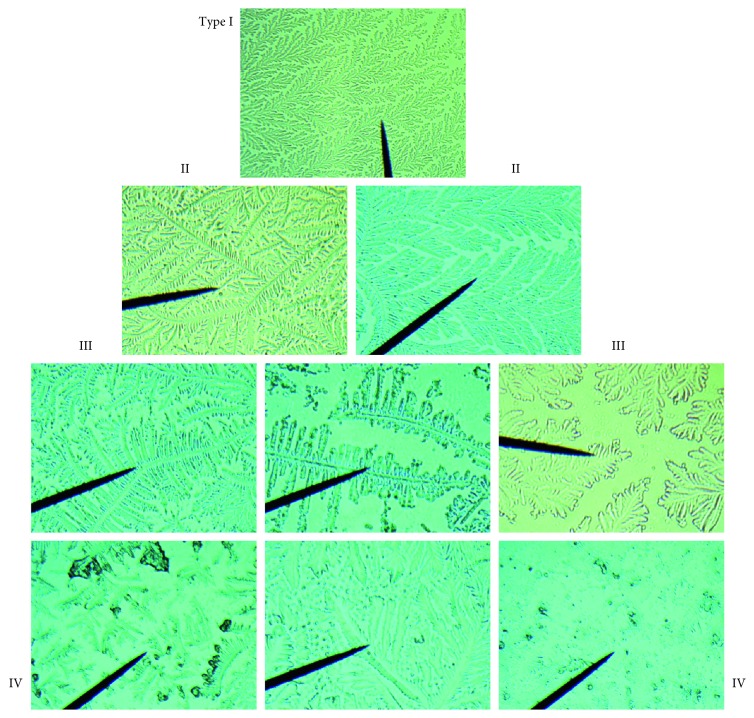
Tear ferns (100×magnification). Type I (control): uniform and compact branched fern. Type II: the number of branches decreased, while the interval increased. Type III: the number of branches decreased significantly, and the interval space increased significantly. Type IV: the number of ferns is low, and the pattern is indefinite.

All patients and controls were subjected to TFT. The technique was performed by drawing tears from the lower marginal lacrimal meniscus with a glass microcapillary tube. The collection of the material was performed at the beginning of the ophthalmic examination to avoid any contamination or dilution by topical anesthetics or colorants such as fluorescein. The sample thus obtained was placed on an optical microscopy slide and left to dry by evaporation at room temperature; slides were then evaluated at the optical microscope to classify the response to the sifting test [13–15]. Thus, qualitative TFT was used for assessment of protein denaturation according to some authors [13–15] (Figure 1).

 
*Type I*, continuous. Large, uniform, and thickly branched ferns without the presence of free spaces between the ferns for the right amount of protein.
 
*Type II*, discontinuous. Abundant felcization but with empty spaces between branches; decreased film stability.
 
*Type III*, reduced. Sparse ferns with empty spaces and mucin accumulations.
 
*Type IV*, no ferns, presence of clusters of degenerate substances. Typical signs of dry eye. Tear film altered by stability, pH, firmness, and osmolarity.

Type I was considered normal to indicate good mucous efficiency and hence indirectly good tear film. Types II and III were considered as a transition form to indicate a state of difficulty in the mucus to maintain its integrity and functions. In types II and III, it is possible to find needle-like ferns instead of arboriforms due to the presence of an abundant protein mat rich in sugars and salts which alters the fern structure. Precipitated glycoproteins are antibodies such as IgM, IgG, and IgE, and in allergic subjects, IgE takes on the appearance of a small cross. Type IV was indicated as corresponding to a profound mucosal alteration. Qualitative TFT was evaluated with a score of 1 to 4 and calculated as type I to IV in both patients and controls, before and after topical therapy.

#### 2.1.2. Conjunctival Impression Cytology

The patients were also subjected to conjunctival impression cytology, taking four samples per patient, two specimens per eye, at the level of the nasal and temporal conjunctiva. Impression cytology was obtained after the administration of topical anaesthesia with 2 drops of 4% ossibuprocain. The filters used for the collection were special nitrocellulose filters (Millicell-®CM Culture Plate Insert, 0.4 *μ*m, Ø 12 mm, PICM 012 50, Millipore). The operator kept the membrane on the conjunctiva for 5 seconds using the plastic support.

The preparations were then fixed with the Bio-Fix cytological fixator (Kaltek s.r.l.), placed in its original container and numbered for subject, eye, area, and date of exam. The samples were stored in a freezer at −80°C, pending semi-Schiff (PAS) staining.

PAS colouration was performed by applying the following procedure to all cytology samples:

- periodic acid at 0.5% for 2 min; washing in distilled water
- Schiff reagent for 10 min; washing in running water trays for 5 minutes
- Pure haematoxylin for 2 min; washing in running water trays for 5 minutes

The samples were then observed under NIKON Eclipse E600 optical microscope to perform caliciform-forming cell counts. The density of the goblet cells was defined as the average of the cells per field in 3 different random fields.

#### 2.1.3. Dosing of MUC5AC Levels

The tears of the patients were collected by inserting Microsponges (Alcon Laboratory, Fort Worth, Texas) into the inferior tarsal conjunctiva of both eyes for 60 seconds. The sponges were removed and put in a 1.5-mL Eppendorf vial containing the subject's identification number, the eye examined, and the date of withdrawal. So prepared, the sample was centrifuged, 12000 revolutions per minute (rpm) for 5 minutes, and stored in a freezer at −80°C. The immunoenzymatic test (ELISA) was performed on the samples collected to search for MUC5AC (Abbexa). The exam was a direct Sandwich test. The tear sample diluted at 30 : 1 was added to prefilled wells with the anti-MUC5AC antibody and left to incubate. A second antibody conjugated to an enzyme such as peroxidase was used to observe the positivity of the reaction. After washing to remove excess substances, the chromogenic substrate, tetramethylbenzidine, was added. Then, a colourimetric reaction was developed to allow quantification of the reagent indirectly bound to the enzyme by means of a suitable spectrophotometer.

### 2.2. Statistical Analysis

Qualitative variables were summarised by percentages and counts. The differences between groups were evaluated by Fisher's test or chi-square test. Quantitative variables were summarised by mean and standard deviation (SD). The differences between the groups were evaluated by nonparametric test by Mann–Whitney. TFT scores, the number of conjunctival goblet cells, and lacrimal MUC5AC levels were compared at baseline and after therapy in the different groups by the *t*-test for independent samples and the Wilcoxon test for paired samples. The correlations between clinical, demographic, and biological parameters were performed by Spearman rho. Values of *p* < 0.05 were considered statistically significant. Statistical analysis was conducted with the SPSS 18.0 software (Statistical Package of Social Sciences, Chicago, IL, USA).

## 3. Results

The study included 47 patients (38 males, 9 females) aged 3 to 16 (mean age 8.8 ± 4.7 SD years) with VKC, and 30 healthy, nonallergic controls (20 males, 10 females), aged 5 to 16 (mean age 11.3 ± 4.2 SD years). According to the literature, VKC prevalence was higher in males than in females (79.85% vs 20.15%) in the population we studied. Fifty-seven percent of patients had a family history of allergic diseases, and 12% had a parent with autoimmune disease. Two patients had VKC in the family (cousin/father). Forty-six percent of the patients were positive for the allergen SPT. Thirty percent of the sample tested had a positive allergic history (25% rhinitis; 5% atopic dermatitis). The average onset of VKC was 2.7 ± 1.3 SD years before. Thirty patients (63.83%) were affected by tarsal form, 12 (25.53%) by limbal form, 3 (6.38%) by mixed form, and 2 (4.26%) by corneal form. The VKC patients instilled 1% CY in 0.1% sodium hyaluronate 3 times at day. Then, they were re-evaluated 3 months after the start of topical therapy. The clinical and demographic characteristics of the sample being studied are summarised in Table 1.

The mean ages between the different groups were not statistically significant. The test results were found to be normal in the control group and abnormal in the VKC group.

**Figure 2:**
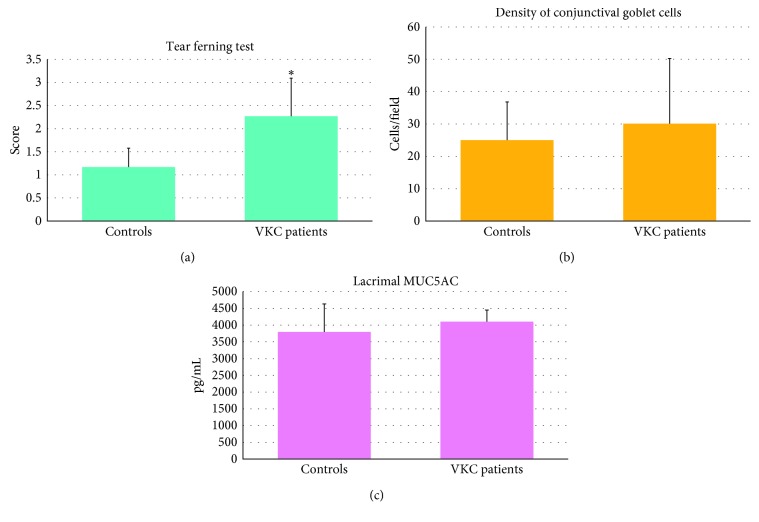
Significant increase of the TFT (tear ferning test) score in VKC (vernal keratoconjunctivitis) patients, 2.43 ± 0.80 SD, vs. controls, 1.17 ± 0.40 SD (*p* < 0.001) (a). Increase in the goblet cells density, though not statistically significant, 30 ± 20 SD cells/field in VKC patients, vs. controls, 25 ± 11 SD cells/field (b). The concentration of MUC5AC does not show significant differences between VKC patients, 4,103.33 ± 286.35 SD pg/mL, and controls, 3,795.40 ± 837.44 SD pg/mL (c).

Comparing the VKC group vs. healthy controls at baseline, we found a significant increase in the degree of the TFT that was 2.43 ± 0.80 SD in VKC subjects and 1.17 ± 0.40 SD in healthy subjects (*p* < 0.001) (Figure 2). This indicated a pathological alteration of the lacrimal mucous layer. In addition, VKC patients had an increased number of goblet cells, 30 ± 20 SD cells/field, compared to healthy controls, 25 ± 11 SD cells/field, although this was not statistically significant (Figure 2). The concentration of MUC5AC did not show significant differences between patients with VKC and healthy controls: VKC: 4,103.33 ± 286.35 SD pg/mL vs. healthy: 3,795.40 ± 837.44 SD pg/mL (Figure 2).

Probably, this indicates a pathological alteration of the lacrimal mucous layer related to the production of fibroblastic material. An irritation of the goblet cells without statistically significant variation of their number and of the concentration of MUC5AC is present.

**Figure 3:**
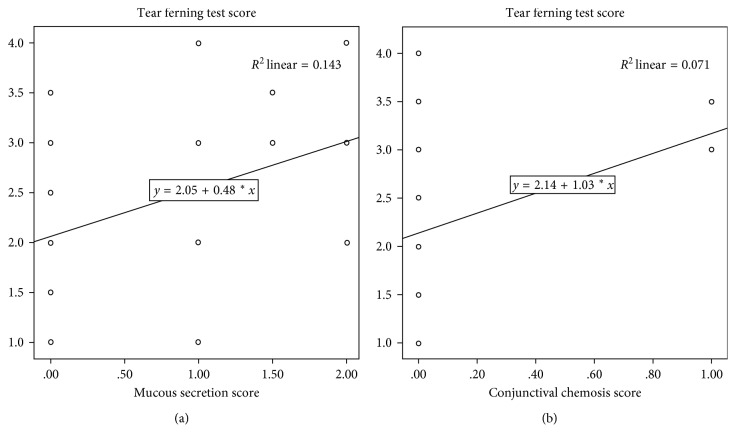
The tear ferning test (TFT) score shows direct correlation with mucous secretion severity (*p*=0.004, *R* = 0.373) (a) and conjunctival chemosis (*p*=0.031, *R* = 0.278) (b), indicating altered quality of the lacrimal mucous component.

By correlating the clinical and biological parameters, the TFT score showed a direct correlation with mucous secretion severity (*p*=0.004, *R* = 0.373) and conjunctival chemosis (*p*=0.031, *R* = 0.278), indicating that in patients with secretion and severe chemosis, TFT also demonstrates poor quality of the lacrimal mucous component (Figures 3 and 3).

**Figure 4:**
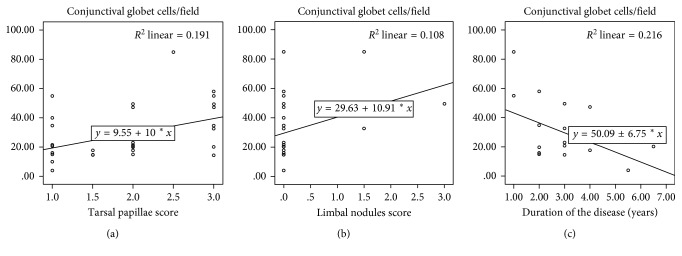
The greatest number of conjunctival goblet cells correlates significantly with the greater severity of the tarsal papillae (*p*=0.005, *R* = 0.499) (a), and limbal nodules (*p*=0.047, *R* = 0.328) (b). In addition, inverse correlation between the number of goblet cells and the duration of the chronic disease was demonstrated (*p*=0.048, *R* = −0.465) for probable chronic damage to the conjunctival tissue (c).

The greatest number of conjunctival goblet cells correlated significantly with the greater severity of the tarsal papillae (*p*=0.005, *R* = 0.499) and limbal nodules (*p*=0.047, *R* = 0.328). In addition, a statistically significant inverse correlation between the number of goblet cells and the duration of the chronic disease was demonstrated (*p*=0.048, *R* = −0.465) for probable chronic damage to the conjunctival tissue (Figures 4–4).

**Figure 5:**
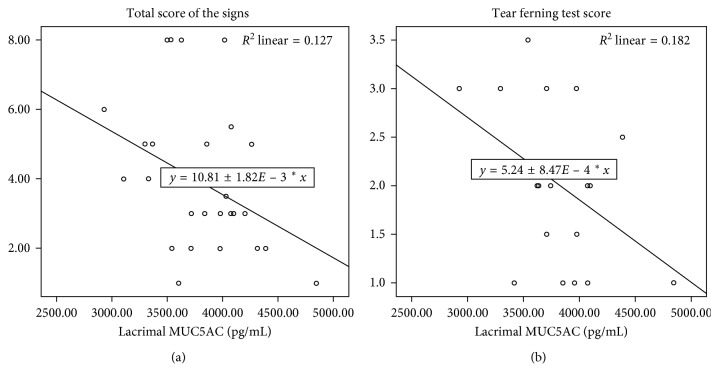
Lacrimal MUC5AC levels correlates inversely with TSS (total score of the signs) (*p*=0.042, *R* = −0.356) (a) and with TFT (tear ferning test) score (*p*=0.047, *R* = −0.427) (b) of VKC (vernal keratoconjunctivitis) patients for less severe VKC and better quality of the tear mucous component.

The lacrimal MUC5AC levels correlated inversely with TSS (*p*=0.042, *R* = −0.356) (Figure 5) and with TFT score (*p*=0.047, *R* = −0.427) (Figure 5) of VKC patients. These data indicated that a higher concentration of MUC5AC correlated with a less severe VKC and with a better quality of the tear mucous component.

**Figure 6:**
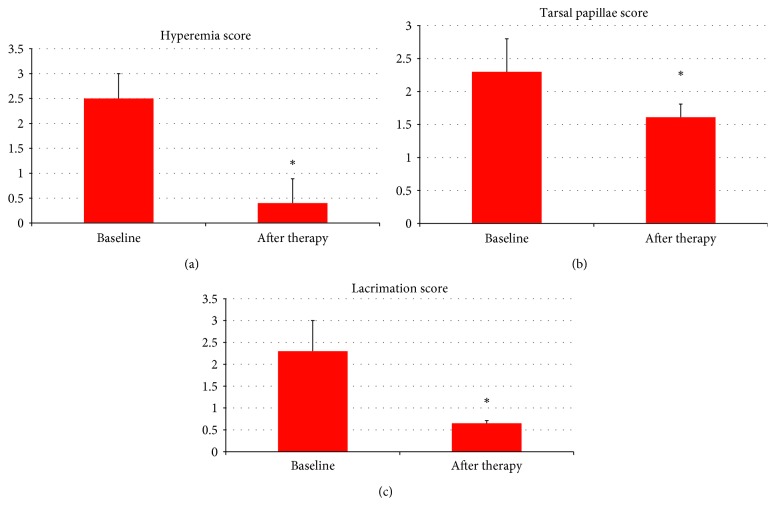
Some signs of pathology improvement 3 months after the start of CY (cyclosporine) therapy, such as: conjunctival hyperemia/chemosis, baseline 2.5 (±0.5 SD) score, and posttherapy 0.40 (±0.49 SD) score (*p*=0.001) (a); tarsal papillae, baseline 2.3 (±0.5 SD) score, and posttherapy 1.61 (±0.2) score (*p*=0.016) (b); and lacrimation, baseline 2.3 (±0.7 SD) score, and posttherapy 0.65 (±0.06 SD) score (*p*=0.045) (c).

**Figure 7:**
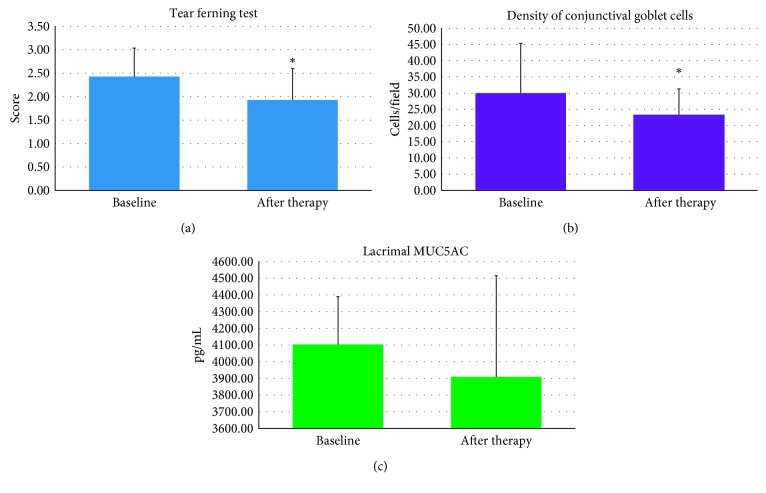
Some signs of pathology improvement 3 months after the start of CY (cyclosporine) therapy, such as TFT (tear ferning test), baseline 2.43 (±0.80 SD) score, and posttherapy 1.93 (±0.67) score (*p*=0.044) (a); density of conjunctival goblet cells, baseline 30 (±20 SD) cells/field, and posttherapy 23 (±8 SD) cells/field (*p*=0.044) (b). There are no changes in the MUC5AC levels before (4,103.33 ± 286.35 SD pg/ml) and after therapy (3,909.18 ± 606.01 SD pg/ml) (c), even because a 3-month treatment period is too short to evaluate the possible functional improvement of the conjunctiva in patients with chronic VKC (vernal keratoconjunctivitis).

Patients were re-evaluated 3 months after the start of 1% CY topical therapy, and no side effect attributable to the use of topical drug was found (Figures 6–6 and 7–7) Some signs and symptoms of pathological activity improved after CY if compared to baseline, such as the following:

1. Conjunctival hyperemia/chemosis, baseline 2.5 (±0.5 SD) score, and posttherapy 0.40 (±0.49 SD) score (*p*=0.001)
2. Tarsal papillae, baseline 2.3 (±0.5 SD) score, and posttherapy 1.61(±0.2 SD) score (*p*=0.016)
3. Lacrimation, baseline 2.3 (±0.7 SD) score, and posttherapy 0.65 (±0.06 SD) score (*p*=0.045)
4. TFT, baseline 2.43 (±0.80 SD) score, and posttherapy 1.93 (±0.67) score (*p*=0.044)
5. Improved density of conjunctival goblet cells, baseline 30 (±20 SD) cells/field, and posttherapy 23 (±8 SD) cells/field (*p*=0.044)

There were no changes in lacrimal MUC5AC levels, baseline 4,103.33 (±286.35 SD) pg/mL, and posttherapy 3,909.18 (±606.01 SD) pg/mL.

## 4. Discussion

In our study, we evaluated the clinical and structural modifications of the lacrimal mucinic component in patients affected by VKC. In particular, the results obtained demonstrated for the first time that there was a significant alteration of the lacrimal mucus in subjects with VKC compared to controls, and moreover, the degree of alteration of the lacrimal mucin component correlated significantly with the severity of the pathology. In addition, the parameters of the disease showed a significant improvement after immunomodulatory therapy with topical CY.

Tabbara and Okumoto were the first to report the use of ferning as a qualitative test for ocular tear discomfort in 91% of patients with several forms of conjunctivitis [16]. Beside, Norn (1994) believes, after having examined for various ocular disorders 225 subjects, that the ferning method has a sensitivity and a specificity of the same order as the commonly used tests for qualitative and quantitative lacrimal film (Schirmer I, BUT, rose bengale, lactoferrin) [15]. Other authors suggest that TFT could be used as a simple, inexpensive, and low-invasive test to clinically evaluate the tear film [13]. Both the BUT and TFT tests reflect the dysfunction of tear mucin indirectly [17]. Our research shows that TFT could be useful in the objective evaluation of tear film and also be a marker of therapeutic efficacy in patients with VKC. Indeed, there are some factors that may change TFT, and the exact nature of what determines the pattern is still not fully understood [13]. In fact, rapid crystallisation of the tears, evaporation, temperature, humidity, impurities, role of electrolytes, spatial location of organic molecules, etc. play a role in the formation of the ferns under the microscope [13]. Precisely for these reasons, according to other authors, it could be more useful to use other tests like counting the number of goblet cells, or rose bengal 1% stain, or lissamine green 2% stain score [18, 19]. For now, the recent literature (Zeev, 2014) asserts that the numerous diagnostic tests used are not yet widely accepted and are often not reproducible [20].

**Figure 8:**
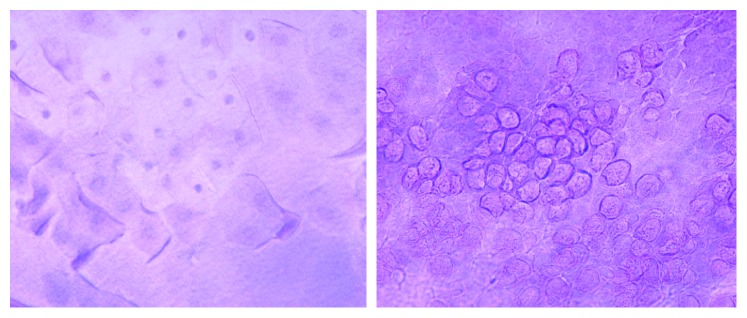
Observation of conjunctival cytogenes by an optical microscope. The goblet cells appear to be clustered and clotted in mucine clusters associated with the presence of metaplasic cellular specimens with some pyknotic nuclei.

Data of our research were accompanied by the demonstration of an increased density of goblet cells in the conjunctival epithelium of VKC patients with a more severe score of tarsal and limbal papillae, both of which are indexes of disease severity [21]. Aragona et al. demonstrated that, in the conjunctival epithelium of VKC patients, there were alterations in calcium density, intercellular junctions, the amount of nuclear chromatin, and the degree of cellular keratinization with a higher number of goblet cells, but smaller in shape than in healthy controls [22]. In addition, we observed under optical microscope that the goblet cells appeared to be clustered and sometimes clotted in mucine clusters associated with the presence of metaplasic cellular specimens with some pyknotic nuclei [22] (Figure 8).

Conjunctival epithelium, therefore, reacts to the inflammatory stimulus with structural changes that can be considered partly as defense mechanisms and partly as degenerative signs. On the contrary, in both type-1, IgE-mediated, and type-4 hypersensitivity reactions, T-lymphocytes play a role in the development of VKC disease. Subsequently, the increase in mucus secretion could also play an important role in the pathogenesis of the disease by providing a network on which allergens may bind, stressing inflammatory stimulation. This could indicate a pathological alteration of the lacrimal mucous layer related to infiltration of conjunctival, limbal, and corneal inflammatory cells with production of fibroblastic material. In fact, the conjunctiva is characterised by an infiltration of inflammatory cells, especially eosinophils, mast cells, and T-lymphocytes [3, 21–23]. Patients with VKC have been shown to have increased levels of activated CD4+, Th2-lymphocites, and of inflammatory cytokines IL-3, IL-4, IL-5, and IL-6, indicating that there is a hypersensitivity reaction to an unknown pathogen [3, 21–23]. Inflammatory cytokines may regulate the secretion, proliferation, and apoptosis of goblet cells [24–26], while allergic mediators such as histamine, leukotrienes, and prostaglandins directly stimulate the secretory activity of these cells [24–27].

Our patients showed only an irritation of the goblet cells without a statistically significant variation of their number and of MUC5AC concentration. This aspect would represent the first phase of conjunctival inflammatory response. With the persistence of the inflammatory stimulus, the degenerative cell phenomena would manifest themselves as the sign of persistent damage to the ocular surface [22].

Moreover, we showed that, in patients with long-term disease, the density of conjuntival goblet cells is lower (Figure 4), indicating a progressive squamous metaplasia of the conjunctiva associated with the duration of chronic inflammation. This could be due to the fact that inflammatory mediators initially stimulate a hyperplasia of conjunctival goblet cells while the persistence of the inflammation could cause a reduction in the density of goblet cells [21–23]. In our study, we also evaluated the tendency of the number of conjunctival goblet cells to normalise in patients with VKC after topical CY therapy. Although these data do not reach statistical significance, they stimulate a reflection on the immunoregulatory properties of drug treatment. Therapeutic activity could be useful to stimulate the still viable cells that in just 3 months of topical therapy have recovered their functionality [28, 29].

As is well known, CY is an immunomodulator that reduces the proliferation of CD4+ lymphocytes through interleukin-2 (IL-2) transcription blockade; it reduces conjunctival fibroblasts and IL-1 production [2, 30, 31]. CY also has an inhibitory effect on the activation of eosinophils, basophils, and mast cells and on the release of histamine, and inflammation mediators [30, 32]. We noticed a significant improvement in the signs and symptoms of patients with VKC after 3 months of 1% CY topical therapy [32, 33]. Our data did not reveal differences in the concentration of lacrimal MUC5AC between patients with VKC and healthy controls, but correlations with clinical data showed a greater concentration of MUC5AC in the tears of less severe patients and with a better quality of mucus (Figures 5 and 5). Such evidence suggests a protective role of MUC5AC, where a higher concentration of lacrimal MUC5AC is associated with better quality of the tear mucus component. In fact, we know that MUC5AC contributes to making the tear film a non-Newtonian viscoelastic structure, capable of modifying its viscosity depending on the size of the applied force, thereby reducing the friction between the eye and eyelid [24, 27].

## 5. Conclusions

This research confirmed the data of the literature on efficacy of topical CY therapy in patients with VKC, demonstrating a statistically significant improvement in clinical and structural ocular inflammation. The results also showed, for the first time, a significant alteration of the lacrimal mucinic component evaluated by TFT in subjects with VKC compared with healthy controls, before and after CY topical therapy. These observations encourage to consider a possible use of TFT in daily clinical practice, and the density of conjunctival goblet cells in VKC patients could be a marker of duration of the disease.

A larger study population and an increase in observation time by at least 12 months after therapy will be necessary to better understand the role of mucins in maintaining the integrity of the mucus barrier and of the ocular surface. All this, in order to lead to a therapeutic strategy to reduce the signs and symptoms, avoid conjunctival fibrosis, and allow, when possible, healing from the disease.

## Figures and Tables

**Table 1:** Clinical and demographic characteristics of 47 VKC patients.

Age range	3–16 years
Age (average ± SD)	8.8 ± 4.7 years
VKC onset (average ± SD)	2.7 ± 1.3 years
Sex: male and female	38 (79.85%) 9 (20.15%)
Forms of VKC	Tarsal: 30 (63.83%)
	Limbal: 12 (25.53%)
	Mixed: 3 (6.38%)
	Corneal: 2 (4.26%)
Total score of signs	12.5 ± 2.0 SD
Total score of symptoms	8.1 ± 2.1 SD
TFT score (average ± SD)	2.43 ± 0.80
Lacrimal MUC5AC (pg/ml)	4,103.33 ± 286.35 SD
Density goblet (cells/field)	30.0 ± 20.0 SD

*Score of signs (TSS)*	*Average ± SD*
Hyperemia/chemosis conjunctival	2.5 ± 0.5
Mucous and serious secretions	2.3 ± 0.7
Tarsal papillae < 3 mm	2.3 ± 0.5
Tarsal papillae > 3 mm	2.2 ± 0.4
Horner–Trantas nodules	2.5 ± 0.2
Epithelial keratitis	0.5 ± 0.02

*Score of symptoms (TSyS)*	*Average ± SD*
Itching and photophobia	4.0 ± 0.7
Tearing	2.1 ± 0.8
Foreign body sensation	2.0 ± 0.6

VKC: vernal keratoconjunctivitis; SD: standard deviation; TFT: tear ferning test.

## Data Availability

The data of this study are in the medical database of Policlinico Umberto I, Sapienza University of Rome.
